# Author Correction: Interrupting oral infection of *Porphyromonas gingivalis* with anti-FimA antibody attenuates bacterial dissemination to the arthritic joint and improves experimental arthritis

**DOI:** 10.1038/s12276-018-0140-z

**Published:** 2018-08-29

**Authors:** Sang Hoon Jeong, Yoojun Nam, Hyerin Jung, Juryun Kim, Yeri Alice Rim, Narae Park, Kijun Lee, Seungjin Choi, Yeonsue Jang, Yena Kim, Ji-Hoi Moon, Seung Min Jung, Sung-Hwan Park, Ji Hyeon Ju

**Affiliations:** 1 Division of Rheumatology, Department of Internal Medicine, Seoul St. Mary’s Hospital, College of Medicine, The Catholic University of Korea, Seoul, South Korea; 2 Department of Maxillofacial Biomedical Engineering, School of Dentistry, and Department of Life and Nanopharmaceutical Sciences, Kyung Hee University, Seoul, South Korea

**Correction to**: *Exp. Mol. Med.* **50**, e460 (2018). 10.1038/emm.2017.301; Published Online 23 March 2018.

After online publication of this article, the authors noticed an error in the Figure section. The correct statement of this article should have read as below.

In the article cited above, incorrect figure was placed in Fig. 5a.

**Fig. 5:**
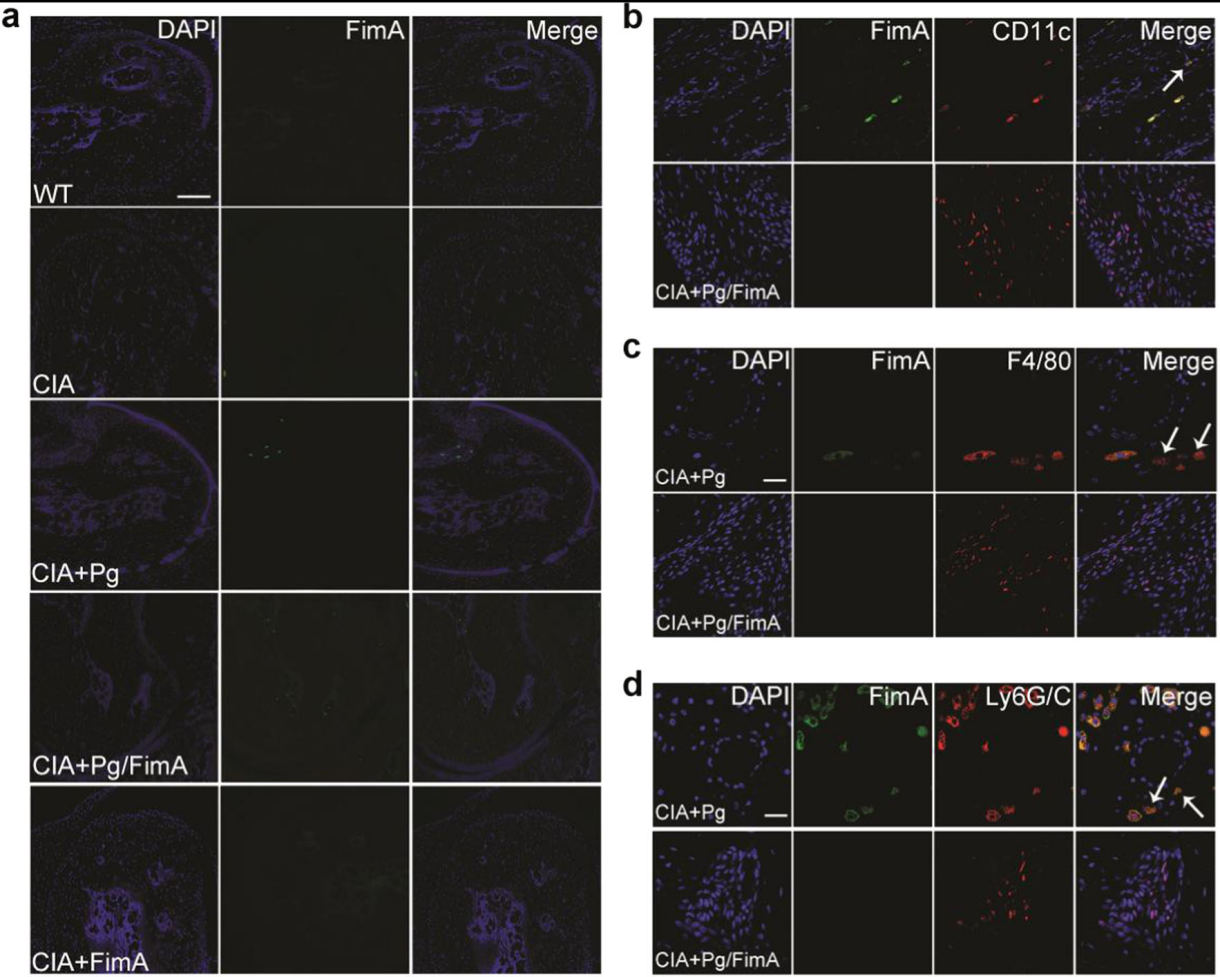
▓

The corrected material is printed below. Other parts of this article remain unchanged. The authors apologize for any inconveniences they may have caused.

